# Detection and assessment of postoperative pain in children with cognitive impairment: A systematic literature review and meta‐analysis

**DOI:** 10.1002/ejp.1936

**Published:** 2022-03-17

**Authors:** Anna Pizzinato, Ilaria Liguoro, Anna Pusiol, Paola Cogo, Alvisa Palese, Enrico Vidal

**Affiliations:** ^1^ Department of Medicine (DAME) School of Nursing University of Udine Udine Italy; ^2^ Department of Medicine (DAME) Division of Pediatrics University of Udine Udine Italy

## Abstract

**Background and Objective:**

Children with cognitive impairment (CI) are at risk of experiencing pain. Several specific pain rating scales have been developed to date. Thus, the aim of this meta‐analysis was to estimate the degree of reliability of different pain assessment scales for the postoperative pain in children with CI.

**Databases and Data Treatment:**

PubMed, Scopus and Web of Science databases were approached: all studies validating and/or using pain assessment tool in children (0–20 years) with CI published in English from the 1st of January 2000 to the 1st of January 2022 were included. Only studies reporting the interclass correlation coefficient (ICC) to evaluate the concordance between caregivers’ and external researchers’ scores were eligible.

**Results:**

Twelve studies were included (586 children with CI, 60% males; weighted mean age 9.9 years – range 2–20). Five of them evaluated the Non‐Communicating Children's Pain Checklist‐Postoperative Version (NCCPC‐PV) scale whereas four the original and revised Face, Legs, Activity, Cry, Consolability (FLACC) scale. The analysis showed an overall ICC value of 0.76 (0.74–0.78) for the NCCPC‐PV scale, with a high heterogeneity index (*I* ^2^ = 97%) and 0.87 (0.84–0.90) for the FLACC scale, with a discrete *I* ^2^ index (59%).

**Conclusions:**

The NCCPC‐PV and FLACC pain rating scales showed the strongest evidence for validity and reliability for assessing postoperative pain in children with CI. However, due to the high heterogeneity of the studies available, these results should not be considered conclusive.

**Significance:**

This review is focused on the assessment of pain in children with CI in the postoperative period. Simplified observation‐based pain assessment tools that rely on evaluating non‐verbal expressions of pain should be recommended for children with difficulties to communicate their feelings. Even if there is a high degree of heterogeneity in clinical presentations among youth with CI, two tools (NCCPC‐PV and FLACC) have emerged as reliable and valid in this population.

## 1 INTRODUCTION

Children with severe cognitive impairment (CI) have been recognized as being at greater risk of experiencing pain in comparison to healthy controls (Breau et al., 2003) especially during daily care activities (Bourseul et al., 2016; Zernikow et al., 2012). They have been reported also at high risk of chronic conditions (e.g., gastro‐oesophageal reflux, hip luxation, tooth decay) triggering pain (Helen & Burne, 1995) and requiring surgical procedures (e.g., Doralp & Bartlett, 2010). While recent advances in pain assessment and management have minimized the postoperative discomfort in paediatric care (Vittinghoff et al., 2018), children with CI remain under‐recognized and under‐managed in their postoperative pain (e.g., Malviya et al., 2006).

The term CI encompasses a broad spectrum of situations including any intellectual disability or global developmental delay, and describes the condition of a child whose intellectual functioning level and adaptive skills are significantly below the average for a child of his/her chronological age (American Psychiatric Association, 2013). Clinically it is important to distinguish children with CI who are able to report pain from those who are not. Up to 50% of child with borderline or mild to moderate CI have been reported to be able to use the numerical pain‐rating scale (Zabalia, 2013). Therefore, whenever possible, self‐report remains the most useful tool for pain assessment among children with CI (Dubois et al., 2010).

However, CI can be due to a variety of clinical conditions including cerebral palsy, genetic syndromes, traumatic brain injury, neurodegenerative disorders, and there is no agreement regarding which pain assessments tool should be used in the context of postoperative pain. Moreover, among children with autism spectrum disorders (ASD), there is a high heterogeneity in the expression (or in the lack of expression) of pain: patients may present hyper‐ and hyposensitivity to pain up to no overt pain signs (Clarke, 2015). Children with ASD usually show some qualitative impairments in language abilities, expressing pain through stereotyped behaviours that may be misinterpreted (Courtemanche et al., 2016). Early studies in these patients reported no reactions, such as facial expressions nor avoidance reflex, in response to pain; however, increased heart rate and blood endorphin levels were recorded during painful episodes compared to children in the control group (Oberlander et al., 1999). Therefore, the lack of behavioural responsiveness does not mean no pain perception, but rather represents a kind of physiological modality to express it (Ely et al., 2016; Ghai et al., 2008). As a consequence, pain assessment in children with CI should include observations of physiological and behavioural changes potentially related to pain. Family caregivers and/or healthcare professionals are crucial in detecting episodes of pain.

In this context, several pain rating tool, checklists or scales (hereinafter scales) have been developed including various sets of potential pain indicators (Breau et al., 2002; Cascella et al., 2019; Ghai et al., 2008; Johansson et al., 2010; Koh et al., 2004; Massaro et al., 2014; Palese et al., 2021; Zanchi et al., 2017). However, some scales have been created without attention to research literature and include items that have not been validated as pain‐specific; hence, their evidence base is questionable (Barney et al., 2020). To our best knowledge, no systematic reviews and no meta‐analysis have been performed to date in the attempt to assess the most reliable pain scale aiming at supporting clinicians in their practice. Therefore, the main intent of this study was to fill‐in the gap by (a) summarizing the evidence available on scales assessing postoperative pain in children with CI, and (b) identifying the scale with the most reliable properties in this field. We used meta‐analytic methods to cumulate interrater reliabilities across studies and to estimate the degree of reliability of different pain assessment scales.

## 2 METHODS

The systematic review was conducted in compliance with the Preferred Reporting Items for Systematic Reviews and Meta‐analyses guidelines (Page et al., 2021).

### 2.1 Literature search

An electronic search was conducted on studies published in the last twenty years, from the 1^st^ of January 2000 to the 1^st^ of January 2022 in PubMed, Scopus and Web of Science databases. The time frame was defined according to the intent of identifying recent studies validating scales in children with CI who underwent healthcare procedures, embodying the several initiatives aimed at – among others –controlling pain in children (Schechter et al., 2010). In our search strategy, we incorporated the use of MeSH terms in addition to keywords deducted from relevant titles and abstracts of studies in the field in order to narrow the search, as the topic was particularly specific. Therefore, the following terms were combined and applied in the approached database: Population: *autism spectrum disorder*, *cognitive impairment*, *children*; *an*d Exposure/Outcome(s): *pain*, *pain assessment*, *pain management*. Examples of the adopted search strategy are shown in Table S1.

After identifying the list of pain assessment scales used, an additional search was performed by using the emerged scales to check, if any, additional studies had been published. To identify missing studies, we also checked the reference list for each included paper.

### 2.2 Inclusion criteria

The review seeks to include all studies providing data regarding the reliability and the validity of pain assessment scales concerning youths with CI as determined by the use of “cognitive impairment” in the study and/or the specific use of diagnostic inclusion criteria related to ASD, global developmental delay or any other condition (e.g., cerebral palsy, genetic syndrome, perinatal or traumatic brain injury) implying an intellectual disability. Thus, all primary studies with quantitative design structure focusing on (a) pain assessment of children/youth aged from 0 to 20 years (hereinafter, children) with any CI, (b) enrolled in a hospital or an outpatient setting for any surgical/invasive procedure, and (c) published in the English language, were included. Opinion and clinical commentary articles, narrative reviews, as well as single case reports, were excluded.

Studies were first screened by title and abstract: duplicates and those with no available English summary were excluded. Eligible full texts were then assessed for inclusion criteria. Papers reporting information on both children and adults were included only if paediatric data could be retrieved and extracted.

To further ensure the high quality of reporting of this review, included studies were also assessed for methodological quality according to the Joanna Briggs Institute (JBI) Critical Appraisal Tools (Munn et al., 2014) (Table S2).

### 2.3 Data extraction

A standardized grid was developed and then piloted in a preliminary fashion on 3 studies. Then, the final version was used for data extraction regarding: first author, country, date and journal of publication, study design (cohort study, case series, retrospective study, case‐control study), sample size, age, aetiology of CI (if reported), clinical setting, surgical/invasive procedure, pain assessment scale, primary outcome and conclusion(s).

During the data analysis process, studies were also grouped according to the adopted pain assessment scale. Two researchers (A. Piz. and I.L.) extracted the data independently and then they agreed on the findings. A third researcher (A. Pal.) was consulted in case of discordances.

### 2.4 Meta‐analytic process

Meta‐analysis was planned to assess the reliability of the included pain assessment scales. In particular, only studies reporting the Interclass Correlation Coefficient (ICC) to evaluate the concordance between caregivers’ and external researchers’ scores were selected. Therefore, the ICC was considered as a measure of reliability or reproducibility of quantitative measurements as expressed by different observers (Bobak et al., 2018; Shrout & Fleiss, 1979). As secondary outcome, we also evaluated if there were significant differences between caregivers’ and observers’ scores for each assessment scale.

### 2.5 Statistical analysis

A quantitative synthesis of the included studies was performed. For continuous variables, weighted mean (range) was calculated as appropriate, while categorical variables were expressed as percentages or frequencies.

Statistical analysis was performed using the Review Manager 5 software (RevMan 5; The Cochrane Collaboration, Oxford, UK). The heterogeneity of the studies was quantified using the I^2^ statistic, with I^2^ values greater than 50% indicative of substantial heterogeneity (Higgins et al., 2013), and a random‐effects model was applied. The results were represented in a forest plot by comparing for each scale the intraclass classification (ICC) reported by the selected studies. Differences between external researchers’ and caregivers’ scores in means (confidence intervals [CI] at 95%) were also analysed.

## 3 RESULTS

### 3.1 Studies

**FIGURE 1:**
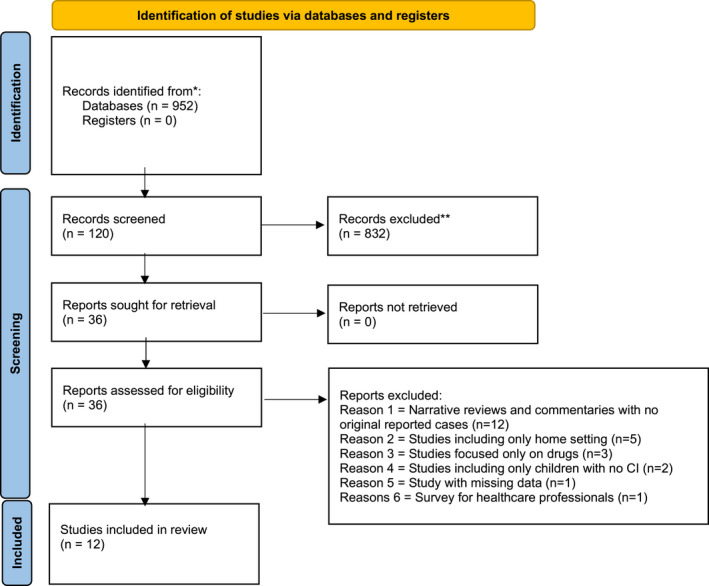
Preferred Reporting Items for Systematic Reviews and Meta‐analyses (PRISMA) 2020 flow diagram of the included studies on pain assessment scales in children with cognitive impairment (CI). Adapted from Page et al. (2021). For more information, visit: http://www.prisma‐statement.org/

A total of 952 papers on the use of pain assessment scales in children with CI were initially identified. After removing duplicates, 120 articles were screened by title and abstract, and subsequently 36 evaluated for eligibility. Finally, 12 studies were included in the analysis (see Figure 1 for details) (Breau et al., 2002; Duivenvoorden et al., 2006; Ely et al., 2016; Johansson et al., 2010; Malviya et al., 2006; Solodiuk et al., 2010; Terstegen et al., 2003; Voepel‐Lewis et al., 2002, 2005, 2008; Zanchi et al., 2017), with a total sample size of 586 children with CI (60% males) and a weighted mean age of 9.9 years (range 2–20). Diagnoses of CI in the enrolled population were mainly represented by cerebral palsy (111/586, 19%), genetic syndromes (57/586, 10%), perinatal complications (44/586, 7.5%), communicating (40/586, 7%) and non‐speaking ASD (22/586, 4%). These patients were evaluated in a hospital/outpatient setting during surgical or minimally invasive procedures (e.g., tooth extraction, percutaneous endoscopic gastrostomy button change, tonsillectomy, vascular accesses positioning). A complete description of the included studies is summarized in Table 1. As reported in Table S2, some studies did not clearly define the inclusion criteria and the period of data collection, as well as omitted details on the population and the setting considered.

**TABLE 1:** Characteristics of the included studies on pain assessment scales and main outcome measures in children with cognitive impairment.

Author	Country	Study design	Study aims	Pain assessment scale(s)	Patients (N)	Age, average (range)	Diagnosis (N)	Setting	Procedures (N)	Measures of outcome	Main conclusions
Breau et al. (2002)	Canada	Diagnostic test accuracy	To validate the NCCPC‐PV scale	NCCPC‐PV *vs* VAS	24	11.5 (3–19)	Genetic syndromes (9), head trauma (3), perinatal complications (5), extreme prematurity (2), neurodegenerative syndromes (1), not known (4)	Day‐surgery	Dental extractions (5); G‐button insertions/removals (3); orthopaedic surgery (3); myringotomy tube insertion (2); heel cord–tendon lengthening (2); venous access device insertion (2); strabismus repair (2); other biopsies or endoscopies (6)	VAS: comparison of pre‐post‐surgery scores; correlation of VAS scores among caregivers; NCCPC‐PV: inter‐rater and intra‐rater reliability; comparison of pre‐post‐surgery scores; correlation with VAS; sensitivity and specificity	NCCPC‐PV is a valid scale to assess pain in children with CI in a post‐procedural hospital setting
Voepel‐Lewis et al. (2002)	USA	Diagnostic test accuracy	To validate the FLACC scale	FLACC *vs* VAS	79	10.1 (4–18)	Children with various degrees of cognitive impairment	Hospital	Orthopaedic or general surgery	Reliability assessed with Cohen's Kappa; validity evaluated with the correlation coefficient	The FLACC tool can be used as an objective measure of postoperative pain in children with CI
Terstegen et al. (2003)	The Netherlands	Cross‐sectional	To identify indicators of postoperative pain in children with CI	138 possible pain indicators *vs* VAS	52	8 (3–19)	Congenital/metabolic syndromes (12), brain abnormalities (12), infections (5), metachromatic leukodystrophy (3), intrauterine‐perinatal asphyxia (9), drowning (1), others (10)	Hospital	Surgery on gastrointestinal tract (27); orthopaedic surgery (11); ENT surgery (9); dentistry (2); plastic surgery (1); cystoscopy (1); enucleation of the eye (1)	The pain indicators that emerged were compared with the scores obtained from the VAS scale	23 pain indicators (PBC) of the 138 possible indicators studied were sensitive to the detection of postoperative pain in children with CI
Voepel‐Lewis et al. (2005)	USA	Diagnostic test accuracy	To assess the validity of caregivers’ FLACC scores	FLACC *vs* 0 to 10 global Numbers pain scores	52	11.3 (4–19)	Cerebral palsy (26), ASD (8), congenital syndromes (9), others (8)	Hospital	Surgery	Reliability evaluated with ICC and with Cohen's Kappa	Parents of children with CI provide reasonable estimates of their child's pain, but tend to overestimate it during the early postoperative period
Duivenvoorden et al., (2006)	The Netherlands	Cross‐sectional	To identify whether the 23‐item version of the PBC can be reduced to 10 items	Reduced PBC	73	6.5 (2–19)	Children with cognitive impairment, varying from severe to profound, i.e., children with an IQ equivalent <35	Hospital	Surgery on gastrointestinal tract (35); orthopaedic surgery (14); ENT surgery (15); dentistry (5); plastic surgery (1); cystoscopy (2); enucleation of the eye (1)	Reduced PBC results compared with the dichotomized VAS results (<4/>4); reliability assessed with Mokken model	The 10‐item version of PBC is a reliable tool for assessing postoperative pain in children with cognitive disabilities
Malviya et al. (2006)	USA	Diagnostic test accuracy	To validate the FLACC scale	r‐FLACC *vs* NAPI	52	11.3 (4–19)	Cerebral palsy (26), ASD (8), syndromes with CI (9), others (8)	Hospital	NR	Reliability evaluated with Cohen's Kappa and ICC	The results support the reliability and validity of the r‐FLACC scale as a pain assessment tool for children with CI
Voepel‐Lewis et al. (2008)	USA	Diagnostic test accuracy	To compare three scales	NCCPC‐PV *vs* r‐FLACC *vs* NAPI	52	11.3 (4–19)	Children with various degrees of cognitive impairment	Hospital	Surgery	Reliability of the scales: Cohen's Kappa and ICC; Scale's validity: pain reduction after analgesic administration (r‐FLACC), transition from pain +to pain 0 (for NAPI) and pre‐ and postoperative comparison (NCCPC‐PV). Clinical utility and feasibility of use of each scale with the CUAQ – Cronbach's alpha	Clinicians and nurses who assessed pain in children with CI, reported greater clinical utility due to less complexity and ease of use, for r‐FLACC and NAPI
Johansson et al. (2010)	Sweden	Cross‐sectional	To assess the validity and reliability of the Swedish version of the NCCPC‐PV scale	NCCPC‐PV	32	11 (2–20)	Cerebral palsy (17), genetic syndromes (7), ASD (4), not known (4)	NR	NR	Caregiver *vs* observer; pain *vs* not pain; validity assessed with ICC	The Swedish version of the NCCPC‐PV can be used for pain assessment in children with cognitive disabilities
Solodiuk et al. (2010)	USA	Diagnostic test accuracy	To validate the INRS scale	INRS vs NCCPC‐PV	50	10.4 (6–18)	Pre/perinatal event (30), epilepsy (4), others (16)	Hospital	Spinal fusion (16); other orthopaedic surgeries (26); general surgeries (6); ENT surgeries (2)	Validity assessed with ICC; compared with NCCPC‐PV scale	The INRS scale is a valid and reliable tool for assessing pain in children with noncommunicating intellectual disabilities in the postoperative period
Massaro et al. (2014)	Italy	Cross‐sectional	To compare three scales	NCCPC‐PV *vs* DESS *vs* CHEOPS	40	9.1 (3–18)	Cerebral palsy (22), mitochondrial encephalopathy (4), epileptic encephalopathy (2), genetic syndromes (3), others (9)	Out‐ and in‐hospital	Surgery (13); venipuncture (11); botulinum toxin injection (6); nasogastric tube placement (3); enema (1); infections (5); abdominal pain (1)	Validity assessed with ICC; structured interview to caregivers	The NCCPC‐PV scale was found to be the most adequate and easiest to use according to caregivers
Ely et al. (2016)	USA	Descriptive qualitative	To identify barriers in pain assessment, describe methods of pain expression using familiar vocabulary	iPad applications (doodle app and Body outline); Hester Poker Chip tool; Wong‐Baker FACES pain rating scale; VAS	40	11.7 (6–17)	ASD with verbal communication skills	Hospital	Orthopaedic (17); Gastrointestinal‐related (9); ENT (9); General surgery (2); Plastics (2); Neurosurgery (1)	Structured interviews: answers were coded into words used to describe one's pain, preference for pain assessment method and how to manage it	No pain detection method was preferred; the importance for children of parents’ presence was noted; 75% of participants indicated distraction as a method used to feel less pain
Zanchi et al. (2017)	Italy	Diagnostic test accuracy	To assess the validity and reliability of the Italian version of the NCCPC‐PV scale	NCCPC‐PV	40	9.5 (3–18)	Cerebral palsy (20), genetic syndromes (8), epileptic encephalopathy (5), ASD (2), others (5)	Out‐ and in‐hospital	Venipuncture (30); botulinum toxin (5); surgery for scoliosis correction (4); nasogastric tube insertion (1)	Caregiver vs observer; pain *vs* not pain; validity assessed with ICC	The Italian version of the NCCPC‐PV is a valid and reliable tool and can discriminate episodes of pain and non‐pain in children with intellectual disabilities

Abbreviations: ASD, Autism Spectrum Disorders; CHEOPS, Children's Hospital of Eastern Ontario Pain Scale; CI, Cognitive Impairment; CUAQ, Clinical Utility Attributes Questionnaire; DESS, Echelle Douleur Enfant San Salvador;ENT, Ear Nose and Throat; FLACC, Faces, Legs, Activity, Cry, and Consolability; ICC, Intraclass correlation coefficient; INRS, Individualized Numeric Rating Scale; NAPI, Nursing Assessment of Pain Intensity; NCCPC‐PV, Non‐communicating Children's Pain Checklist–Postoperative Version; NR, Not reported; PBC, Pain Behavior Checklist; VAS, Visual Analogical Scale.

### 3.2 Pain assessment scales

The pain assessment scales used in the included studies were: the Non‐Communicating Children's Pain Checklist‐Postoperative Version (NCCPC‐PV), the original and revised versions of the Face, Legs, Activity, Cry, Consolability scale (FLACC, r‐FLACC), the Nursing Assessment of Pain Intensity scale (NAPI), the Echelle Douleur Enfant San Salvador scale (DESS), the Children's Hospital of Eastern Ontario Pain Scale (CHEOPS), the Pain Behavior Checklist (PBC), with the reduced version, and the Individualized Numeric Rating Scale (INRS). Terstegen et al. (2003) also used a group of 134 possible pain indicator items from which 23 resulted predictive for pain, while Ely et al. (2016) adopted the pain self‐report methodology through the use of tablets with the Doodle and Body Outline applications, the Visual Analogic Scale (VAS) scale and the Wong‐Baker Faces Pain Rating Scale (WBFPRS).

The included studies were then grouped according to the used scale: five (Breau et al., 2002; Johansson et al., 2010; Massaro et al., 2014; Voepel‐Lewis et al., 2008; Zanchi et al., 2017) evaluated the NCCPC‐PV scale, and four (Malviya et al., 2006; Voepel‐Lewis et al., 2002, 2005, 2008) evaluated the original and revised FLACC scale (Table 1).

### 3.3 Meta‐analysis

**FIGURE 2:**
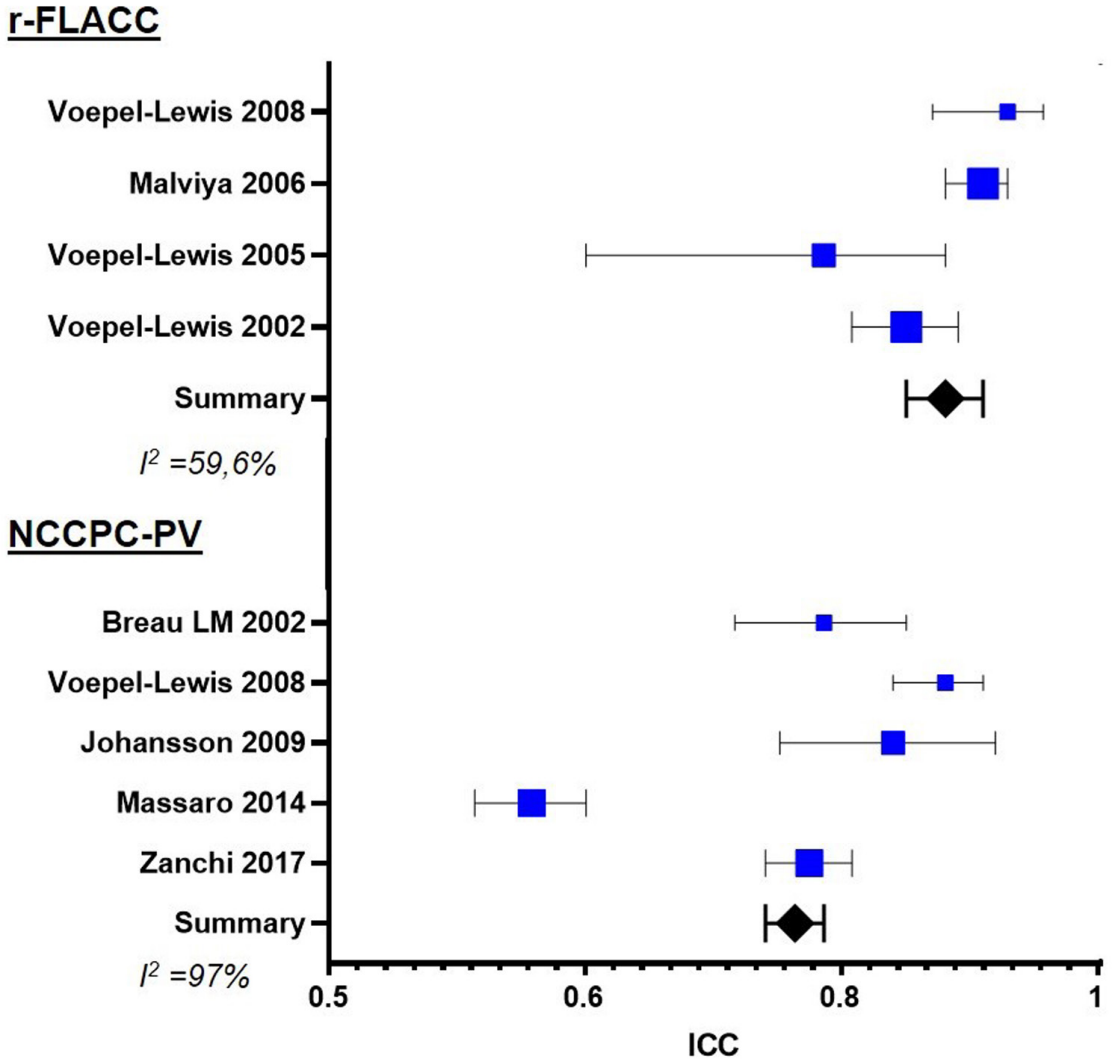
Interclass correlation coefficient (ICC) of the included studies. Non‐Communicating Children's Pain Checklist‐Postoperative Version (NCCPC‐PV); revised version of the Face, Legs, Activity, Cry, Consolability scale (r‐FLACC); heterogeneity index (*I* ^2^).

The individual and cumulative effect size of the pain assessment scale adopted in each study was estimated on the basis of the reliability calculated as ICC. The analysis showed an overall ICC value of 0.76 (0.74–0.78) for the NCCPC‐PV scale, with a high heterogeneity index (*I* ^2^ = 97%) and 0.87 (0.84–0.90) for the FLACC/ r‐FLACC scale, with a discrete *I* ^2^ index (59%) (Figure 2).

**FIGURE 3:**
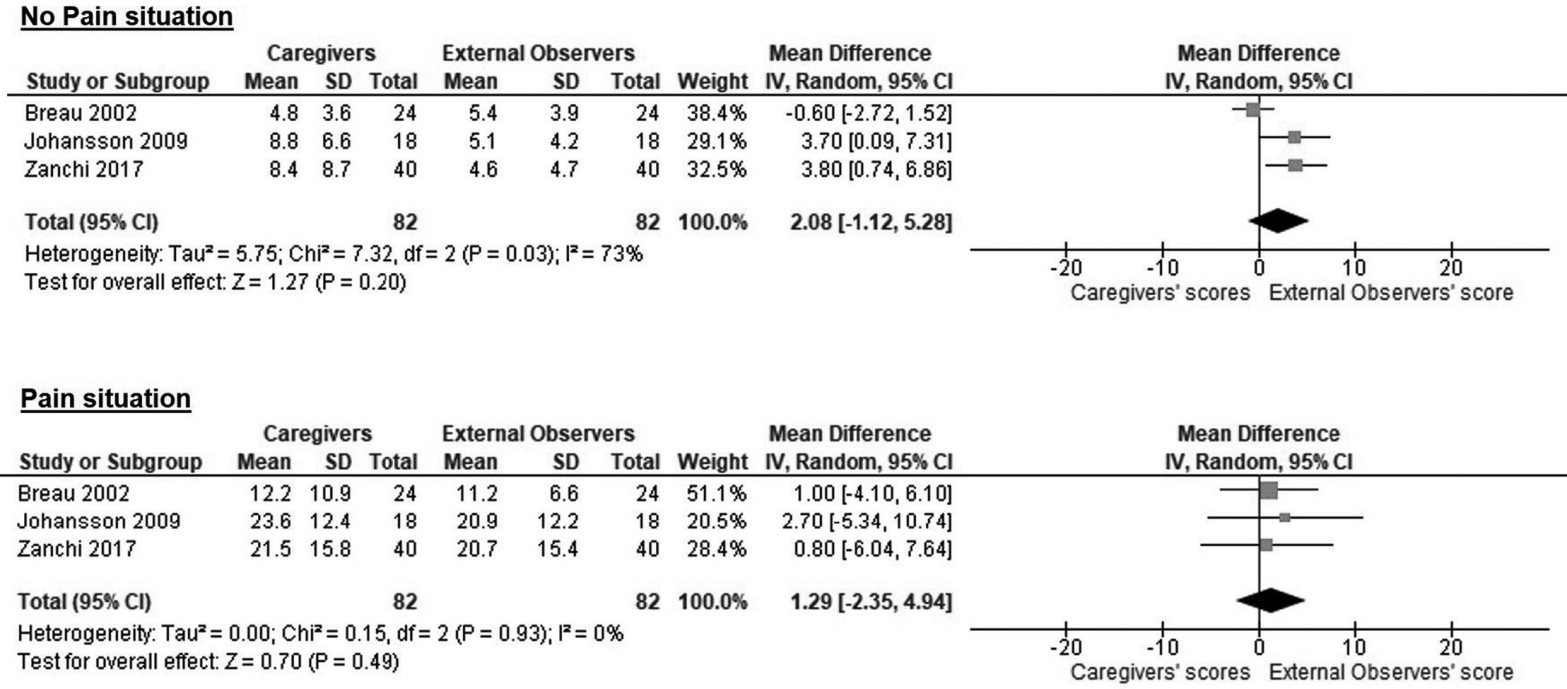
Difference in pain assessment's scores between caregivers and external observers among the included studies for the Non‐Communicating Children's Pain Checklist‐Postoperative Version (NCCPC‐PV). CI, Confidence Interval; IV, Inverse Variance; SD, Standard Deviation.

**FIGURE 4:**
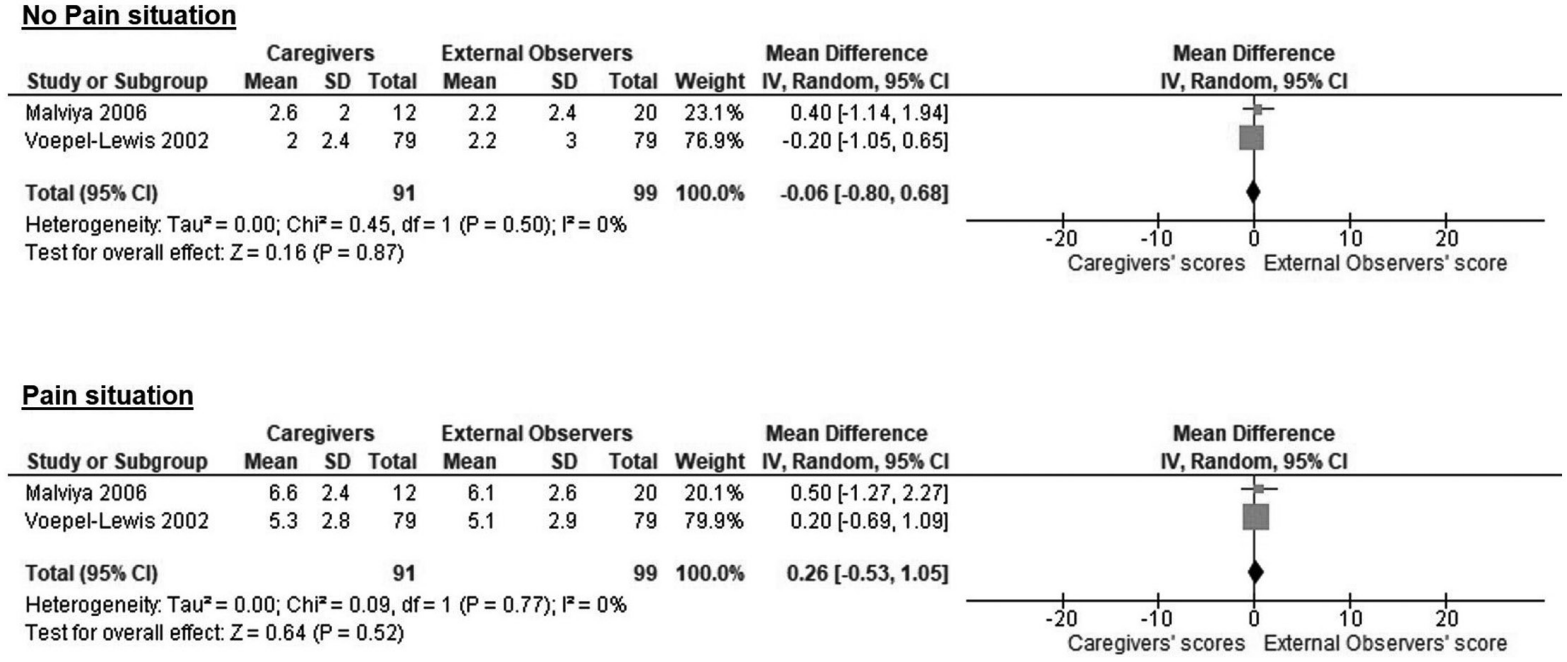
Difference in pain assessment's scores between caregivers and external observers among the included studies for the revised version of the Face, Legs, Activity, Cry, Consolability scale (r‐FLACC). CI, Confidence Interval; IV, Inverse Variance; SD, Standard Deviation.

The NCCPC‐PV and the FLACC scales were then analysed to evaluate any difference in pain assessment between the scores obtained by caregivers and external observers. No statistically significant differences emerged between evaluations from caregivers and researchers for the NCCPC‐PV scale, either in no pain (mean difference 2.08, 95%CI −1.12–5.28; *I* ^2 ^= 73%) and pain situations (mean difference 1.29, 95%CI −2.35–4.94; *I* ^2 ^= 0%) (Figure 3). Similarly, the scores of caregivers and researchers who used the FLACC scale were not significantly different, either in no pain (mean difference −0.06, 95%CI −0.80–0.68; *I* ^2 ^= 0%) and pain situations (mean difference −0.26, 95%CI −0.53 – 1.05; *I* ^2^ = 0%) (Figure 4).

## 4 DISCUSSION

This systematic review followed by a meta‐analysis aimed at reviewing the evidence on the pain assessment scales used in children with CI who underwent surgical procedures. Studies included show several limits in their methodological quality, suggesting the need of improvements in this field of research; according to the lacks emerged, the meta‐analysis based on the ICC, which is an indirect index of reliability (Bobak et al., 2018; Shrout & Fleiss, 1979), might have influenced the findings. Among the identified tools, the NCCPC‐PV and the r‐FLACC scales were the most frequently investigated tools, both showing a high reliability index, but based on very heterogeneous data. The heterogeneity emerged might be interpreted under different lines, as the attempt of researchers to have a pragmatic approach in order to develop and validate tools useful for a range of conditions, mainly for clinical purposes where it might be unfeasible to recommend the use of several scales according to the condition of the child; and as a consequence of the monocentric nature of the studies thus at need to broaden the participants by including a range of conditions and ages in order to achieve the expected sample size.

### 4.1 The NCCPC‐PV

The postoperative version of the NCCPC scale is a 27‐item pain checklist specifically designed for children with CI unable to communicate verbally (Breau et al., 2002). Compared to the original version, the NCCPC‐PV excludes eating/sleeping‐related items, because of the risk to possibly introduce false‐positive results, and a score of 11 or greater resulted able to detect up to 88% of children with clinically significant pain (Breau et al., 2002). Similarly to Johansson et al. (2010) our findings further confirmed that the NCCPC‐PV fairly discriminate painful from not‐painful episodes. We also demonstrated a high inter‐observer reliability between caregivers and researchers, except for the Social and Body and Limb subscales, likely because caregivers were more familiar with the children's abilities to interact socially and to use their body and limbs voluntarily (Breau et al., 2002). The NCCPC‐PV was validated in a Swedish (Johansson et al., 2010) and an Italian (Zanchi et al., 2017) cohort, both showing a good agreement between the different raters in defining the presence or absence of pain, in particular in the painful situations (Johansson et al., 2010; Zanchi et al., 2017). However, the restricted use of this scale to the severely impaired children may also limit the ability to generalize these findings to other populations of children with mild‐moderate CI (Malviya et al., 2006).

According to some authors, the interpretation of the NCCPC‐PV scoring appeared to be inconsistent with other clinical pain measures, and it was therefore ranked by clinicians as significantly less feasible for clinical practice compared with the r‐FLACC (Voepel‐Lewis et al., 2008). In another study (Massaro et al., 2014), the NCCPC‐PV was defined by observers (both researchers and caregivers) as the easiest tool to use in comparison to the DESS and the CHEOPS, with a higher percentage of caregivers (about 80%) feeling the NCCPC‐PV correctly rated their child's pain more often than the other scales. Recently, a revised version of the NCCPC was also specifically validated for children with ASD (Palese et al., 2021).

### 4.2 The FLACC/r‐FLACC

The FLACC pain scale is a validated behavioural scale that is useful in paediatric patients up to the age of 16 who cannot or will not verbalize the severity of their pain and measures both pain and psychologic distress (Merkel et al., 1997; Nilsson et al., 2008). FLACC pain scale measures both pain and psychologic distress, and has been specifically developed and validated for the evaluation of the immediate postoperative period in healthy children aged from 2 months to 7 years (Merkel et al., 1997). It has been showing high interrater reliability, and it is extensively applied in several settings (post‐surgery or peri‐procedural pain management, emergency department) (Crellin et al., 2018; Nilsson et al., 2008; Redmann et al., 2017). It was also considered particularly suitable for children with different types of CI (Voepel‐Lewis et al., 2003, 2008). The FLACC scale contains five categories, each scoring from 0 to 2 to provide a total score ranging from 0 to 10. However, while measures of agreement between observers were found to be acceptable for comparisons in the Face, Cry and Consolability categories, there was low agreement in the Legs and Activity categories (Voepel‐Lewis et al., 2002), similar to the findings by Breau et al. (2002) for the NCCPC‐PV scale. The r‐FLACC version incorporates several additional behavioural descriptors, including verbal outbursts, tremors, increased spasticity, jerking movements, and respiratory pattern changes. Malviya and colleagues demonstrated an improvement in reliability measures for all categories in comparison to previous studies with the addition of these specific behavioural descriptors (Malviya et al., 2006). Moreover, the r‐FLACC allows the individualization of the scoring in each category, as parents of children with CI provide reasonable estimates of their child's pain, particularly when using a structured pain tool (Voepel‐Lewis et al., 2005). Interestingly, several parents noted that a lack of expression or responsiveness was most indicative of pain in their children. Inclusion of these behaviours may have contributed to the improved reliability and validity of the r‐FLACC pain scores (Malviya et al., 2006).

These findings also suggest that for children with CI, a comprehensive preoperative interview may help to establish baseline and individual behaviour that facilitate accurate pain assessment during the entire postoperative period.

### 4.3 Researchers’ and caregivers’ scoring

The data collected from the included studies were also analysed to highlight possible discrepancies in the use of the NCCPC‐PV and the FLACC scales by researchers and caregivers, both in painful and non‐painful situations. However, no statistically significant differences emerged between caregivers’ and researchers’ evaluations for both scales.

In clinical practice, caregivers are often required to interpret the child's behaviour and its relationship to pain, particularly for those with CI, as they are more familiar with their child's normal behaviour than clinicians (Cascella et al., 2019). Some authors demonstrated that mothers become experts in assessing their child's pain without health professionals’ support (Carter et al., 2017), and healthcare personnel tend to underestimate pain in children (Zhou et al., 2008). On the other hand, caregivers may tend to overestimate symptoms in acute pain conditions and underestimate them in the setting of chronic disease (Matziou et al., 2016). Overall evidence remains inconclusive on this aspect (Voepel‐Lewis et al., 2005), and more research is needed on the individual and situational factors that predict concordance between anecdotal parent report and standardized assessment of child pain.

However, our study documented the overall effect of caregivers’ estimates of children's pain in comparison with external observers (e.g., nurses or physicians), by using the NCCPC‐PV and the FLACC scales. The results showed a very good concordance between evaluations from parents and researchers in all studies included, which were based on consistently homogenous and highly comparable populations.

### 4.4 Other pain assessment scales

Several other pain assessment scales were identified but not included in the analysis, due to an insufficient number of studies available on them. Among these, the NAPI was considered one of the easiest tools to adopt in scoring mild, moderate and severe pain in children with CI, with an excellent reliability (ICC) (Voepel‐Lewis et al., 2008).

The CHEOPS, a scale that was not specifically designed for children with CI, resulted easy to use for caregivers, as it required very little information about their children (Massaro et al., 2014), while the DESS was less simple to use for the observers who were not familiar with children (Massaro et al., 2014).

Solodiuk et al. tried to validate the INRS scale comparing it to the NCCPC‐PV (Solodiuk et al., 2010). The authors found a good inter‐rater agreement (ICC 0.64–0.73) between the INRS and the NCCPC‐PV scores. Moreover, according to the parents participating in the study, the INRS scale also allowed to specifically evaluate and recognize the particular behaviour occurring in their children when they were in pain (Solodiuk et al., 2010).

Duivenvoorden et al. aimed to assess the reliability of the reduced PBC, but it resulted less accurate than the original 23 items version (Duivenvoorden et al., 2006). Terstegen et al. (2003) identified more than 200 pain indicators in children with CI by using semi‐structured interviews to parents and to various specialists. Behaviours not suited to the clinical context were then discarded, obtaining a group of 138 items that were tested in the postoperative period in comparison to the VAS scale, and 23 sensitive indicators were included in the final version of the questionnaire (Terstegen et al., 2003).

Another qualitative study was conducted by Ely et al. who tried to identify barriers to methods of pain assessment and pain expression in children with ASD who were able to communicate verbally (Ely et al., 2016). They used self‐report tools, such as iPad applications, the Wong‐Baker Faces Scale and the VAS and reported that their parents were able to properly understand their pain, sometimes using to look to them for confirmation during the interview (Ely et al., 2016).

All these findings underline the need to individualize the pain assessment for each child with CI. Describing pain may be preferred to the use of a number scale, as facial expressions and body language often do not match pain scores or descriptors of pain intensity. However, further evidence is needed to support the validity data of these tools.

### 4.5 Strengths and limitations of the study

One of the main limitations of this review is represented by the fact that the included studies were only a few, very heterogeneous, with small samples and considerable differences in the age range; moreover, studies lacked in reporting the complete characteristics of the patients (as for example, the Gross Motor Function Classification System data), which are suggested to be described in future papers in order to allow the assessment of external validity of the findings. Studies have been also assessed in their methodology quality without considering the findings of this evaluation as inclusion criteria; however, the methodological evaluation performed might address researchers in covering the gaps in future studies. Furthermore, we did not perform any sensitive analysis (e.g., to estimate the stability of their pooled ICC by removing influential studies that might contribute to heterogeneity) given the limited number of studies included for each scale.

There is also accumulating evidence that racial/ethnic minority youth experience disparities related to pain management, including in postoperative settings (Donaldson et al., 2020). Similarly, we have evidence that children who might be characterized as having language difficulties also experience disparities in pain care (Dixit et al., 2020; Friedlaender et al., 2019). The lack of racial/ethnic diversity in the reviewed studies and possible implications for patients with CI (i.e., compounded risk for disparities) represents another important limitation of studies available, suggesting future areas of research improvements.

However, this is the first meta‐analysis focused on the management of pain in children with CI in the postoperative period.

### 4.6 Conclusions

This initial meta‐analysis focused on the assessment of pain in children with CI in the postoperative period documents the availability of two tools (NCCPC‐PV and FLACC) with strong evidence for reliability and validity in this population. However, due to the high heterogeneity of the published studies, findings emerged should not be considered conclusive, and more targeted studies are recommended. Other pain rating scales such as the DESS, CHEOPS, NAPI, PBC and INRS need to be further investigated as well.

Alongside their validity, in assessing pain, healthcare providers should be trained in the use of the various pain assessment scales, particularly when addressing children with CI. Moreover, children with mild CI should be given the chance to use a self‐report scale if health professionals are sure that they fully comprehend how to use the adopted scale. On the other hand, simplified observation‐based pain assessment tools that rely on evaluating non‐verbal expressions of pain should be recommended for children with difficulties to communicate their feelings, in particular when caregivers or other family members are not present, such as in emergency conditions. Given the high degree of heterogeneity in clinical presentations among youth with CI, an array of evidence‐based strategies is needed to allow for tailored selection and implementation of pain assessment in this population.

## CONFLICT OF INTEREST

All authors have no conflicts of interest to disclose.

## AUTHOR CONTRIBUTIONS

EV and IL conceptualized and designed the study. APi and IL collected and analysed data. APi and IL drafted the first version of the article. APa, EV and APu critically revised the manuscript for important intellectual content. All authors discussed the results and commented on the manuscript. All authors approved the final version of the manuscript.

## Supporting information

Table S1

Table S2
