# Supplementary material for: Detection and assessment of postoperative pain in children with cognitive impairment: A systematic literature review and meta‐analysis
Source: Eur J Pain. 2022 Mar 17;26(5):965–79. doi: 10.1002/ejp.1936 (PMC9311729; doi:10.1002/ejp.1936)
Supplement: Supplementary file 1 — Table S1 [file EJP-26-965-s001.docx]

**SUPPLEMENTARY TABLE 1.** Search strings applied in Pubmed.

| String | Search terms | N° of manuscript retrieved |
| --- | --- | --- |
| #1 | (cognitive impairment* OR cognitive deficit) AND (pain* OR pain assessment* OR pain management*) AND (child* OR pediatric* OR adolescent) AND ( LIMIT-TO ( LANGUAGE, "English")) | 697 |
| #2 | (autism spectrum disorder OR cognitive impairment) AND (pain assessment OR pain management) AND (child*) AND ( LIMIT-TO ( LANGUAGE, "English")) | 249 |
| #3 | (cognitive impairment* OR cognitive deficit) AND (post operative* pain) AND (child*) AND ( LIMIT-TO ( LANGUAGE, "English")) | 6 |
