# Supplementary material for: Detection and assessment of postoperative pain in children with cognitive impairment: A systematic literature review and meta‐analysis
Source: Eur J Pain. 2022 Mar 17;26(5):965–79. doi: 10.1002/ejp.1936 (PMC9311729; doi:10.1002/ejp.1936)
Supplement: Supplementary file 2 — Table S2 [file EJP-26-965-s002.docx]

**SUPPLEMENTARY** **TABLE S2**. Evaluation of the included studies according to the Joanna Briggs Institute (JBI) critical appraisal tool (Munn et al., 2014).

| **Author** | **Type of study** | **Relevant points not met according to JBI evaluation** (Schechter et al., 2010) |
| --- | --- | --- |
| Breau et al., 2002 | Diagnostic test accuracy | Patients’ enrollment period has not been accurately described; missing data; inappropriate exclusion of a patient. |
| Voepel-Lewis et al., 2002 | Diagnostic test accuracy | Patients’ enrollment period has not been described; missing data; not clear criteria for inclusion has been established. |
| Terstegen et al., 2003 | Cross-sectional | Setting has not described in detail. |
| Voepel-Lewis et al., 2005 | Diagnostic test accuracy | Missing data; not clear criteria for inclusion has been established. |
| Duivenvoorden et al., 2006 | Cross-sectional | Study subjects and setting have not described in detail. |
| Malviya et al., 2006 | Diagnostic test accuracy | Patients’ enrollment period has not been described; not clear criteria for inclusion has been established. |
| Voepel-Lewis et al., 2008 | Diagnostic test accuracy | Patients’ enrollment period has not been described; not clear criteria for inclusion has been established. |
| Johansson et al., 2009 | Diagnostic test accuracy | Missing data: complete diagnosis was not available for four children. |
| Solodiuk et al., 2010 | Diagnostic test accuracy/ prospective cohort | - |
| Massaro et al., 2014 | Cross-sectional | - |
| Ely et al., 2016 | Descriptive qualitative | - |
| Zanchi et al., 2017 | Diagnostic test accuracy | Demographic characteristics have not clearly reported. |
